# Heat Dissipation in Flexible Nitride Nanowire Light-Emitting Diodes

**DOI:** 10.3390/nano10112271

**Published:** 2020-11-16

**Authors:** Nan Guan, Nuño Amador-Mendez, Arup Kunti, Andrey Babichev, Subrata Das, Akanksha Kapoor, Noëlle Gogneau, Joël Eymery, François Henri Julien, Christophe Durand, Maria Tchernycheva

**Affiliations:** 1 C2N-CNRS, Université Paris Saclay, 91120 Palaiseau, France; nan.guan@hotmail.com (N.G.); nuno.amador@c2n.upsaclay.fr (N.A.-M.); arup.kunti@universite-paris-saclay.fr (A.K.); noelle.gogneau@c2n.upsaclay.fr (N.G.); francois.julien@c2n.upsaclay.fr (F.H.J.); 2 ITMO University, 197101 Saint Petersburg, Russia; a.babichev@mail.ioffe.ru; 3 Materials Science and Technology Division, CSIR-National Institute for Interdisciplinary Science and Technology, Thiruvananthapuram, Kerala 695019, India; physubrata@gmail.com; 4 Univ. Grenoble Alpes, CEA, IRIG, PHELIQS, NPSC, 38000 Grenoble, France; aku.kapoor19@gmail.com (A.K.); christophe.durand@cea.fr (C.D.); 5 Univ. Grenoble Alpes, CEA, IRIG, MEM, NRS, 38000 Grenoble, France; joel.eymery@cea.fr

**Keywords:** nanowire, LED, InGaN, mechanical flexibility, self-heating

## Abstract

We analyze the thermal behavior of a flexible nanowire (NW) light-emitting diode (LED) operated under different injection conditions. The LED is based on metal–organic vapor-phase deposition (MOCVD)-grown self-assembled InGaN/GaN NWs in a polydimethylsiloxane (PDMS) matrix. Despite the poor thermal conductivity of the polymer, active nitride NWs effectively dissipate heat to the substrate. Therefore, the flexible LED mounted on a copper heat sink can operate under high injection without significant overheating, while the device mounted on a plastic holder showed a 25% higher temperature for the same injected current. The efficiency of the heat dissipation by nitride NWs was further confirmed with finite-element modeling of the temperature distribution in a NW/polymer composite membrane.

## 1. Introduction

Flexible light sources have many appealing applications, such as foldable or rollable lighting, and curved and bendable displays for wearable devices. Deformable light sources are highly demanded in advanced biological research, such as biomedical, biological, and optogenetic studies. In the domain of lighting, the interest for large-area curvilinear light sources that can be integrated on various surfaces of furniture and other parts of an interior space is also growing. Light-emitting diodes (LEDs) based on inorganic materials start to compete with organic emitters in applications requiring mechanical flexibility [1,2,3]. Inorganic LEDs present the advantage of long lifetimes and high brightness. The fabrication of flexible devices from conventional thin-film structures requires additional processing steps to lift-off and microstructure the active layer. To simplify the process, alternative fabrication routes were actively explored based on nanomaterials such as bottom-up nanowires (NWs) [4,5,6,7,8,9,10,11,12,13]. NWs not only have remarkable optoelectronic properties, but they can also withstand high deformations without plastic relaxation [14,15]. These properties motivated the intense research of NW flexible LEDs [4,8,9,10,11,12,13,16]. In most realizations, NWs are embedded in a transparent polymer (e.g., polydimethylsiloxane (PDMS), polyimide, SU-8, or parylene [12,17,18,19,20,21]), which acts as a supporting material to form the active membrane. While III–V material composing NWs has a long lifetime and can withstand high temperatures, the embedding polymer is prone to degradation. Additionally, polymers are characterized by poor thermal conductivity (~1.5 W m^−1^ K^−1^ for PDMS [13] compared with ~200 W m^−1^ K^−1^ for GaN [22]), so self-heating issues can be severe in NW/polymer composite membranes. Therefore, one can question the flexible NW LEDs’ ability to operate at high current. Hence, it is important to study the self-heating problem and related degradation of LEDs under thermal stress.

While the thermal properties of thin-film nitride LEDs were thoroughly studied in the literature [23,24,25,26,27], only a few studies address the thermal behavior of NW LEDs focusing on axial-junction devices [28,29]. To the best of our knowledge, no reports exist on heat dissipation in either radial-junction or flexible nitride NW LEDs. It is not straightforward to perform thermal measurements on NW LEDs. Methods to estimate the junction temperature most widely used in the LED industry are the forward-voltage method, which provides a few degrees of accuracy [30,31,32], or the thermal-resistance method, which yields precision close to ±0.1 °C [33]. However, these methods, widely applied to standard thin-film LEDs, need the precise assessment of the device’s electrical parameters, which is challenging for flexible NW devices based on polymer membranes. Furthermore, calibration and LED measurement are complex and require dedicated equipment, so these methods are not well-suited for flexible NW LEDs. Another widely applied method to probe LED temperature is based on measurement of the peak wavelength shift with temperature [32,34]. It is commonly accepted that accuracy of the peak energy value is about 10% of the emission linewidth, yielding precision of ±24 °C for standard LEDs [28,30,32]. However, it is challenging to determine the peak wavelength in NW-based nitride LEDs due to alloy-broadening effects that strongly degrade the accuracy of the method [30], and because current injection lines are prone to changing with bias, which results in a shift of electroluminescence peak that is not related to junction temperature [35,36]. As a result, the peak wavelength shift method is not well-suited for NW LEDs. Infrared (IR) thermal imaging [37,38,39], widely used in the semiconductor industry [40], appears to be the most appropriate method for assessing the flexible NW LED temperature variations. 

In this context, the present work reports the investigation of the thermal behavior of a flexible LED working at room temperature under different injection conditions. Flexible LEDs are based on metal-organic vapor-phase deposition (MOCVD)-grown NWs in PDMS membranes. Two different LED mountings, one on a plastic holder and the other on a copper heat sink, were analyzed. Despite the poor thermal conductivity of PDMS, active nitride NWs can effectively evacuate heat to the substrate. Therefore, the flexible LED mounted on a copper heat sink could operate under high injection without significant overheating, while the device mounted on a plastic holder showed 25% higher temperature for the same injected current, resulting in progressive current reduction in time. The high heat-evacuation efficiency of nitride NWs was further confirmed with finite-element modeling of the temperature distribution in a NW/polymer composite membrane.

## 2. Materials and Methods 

### 2.1. InGaN/GaN Nanowire Growth 

**Figure 1:**
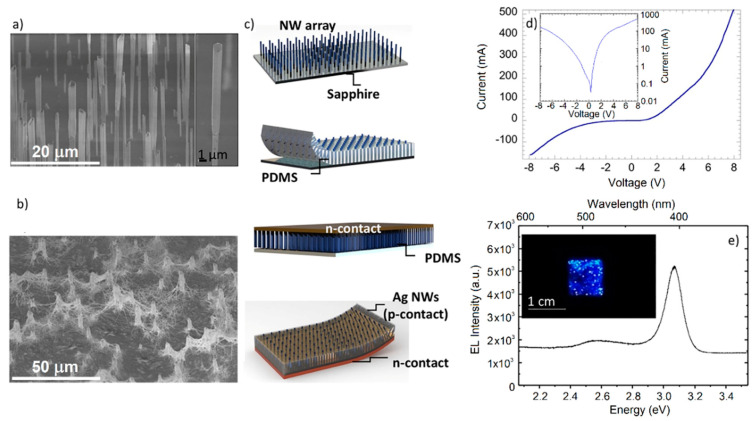
(**a**) SEM image of as-grown sample (inset shows close-up on individual nanowire (NW)); (**b**) SEM image of top contact with protruding InGaN/GaN NWs presenting good coverage by Ag nanowires; (**c**) schematic illustration of main fabrication steps (from top to bottom): as-grown NWs, peel-off of polydimethylsiloxane (PDMS)/NW composite membrane, deposition of bottom metal contact, fully processed light-emitting diode (LED) with top transparent contact; (**d**) current–voltage (I–V) curve of flexible LED. Inset shows I–V curve in logarithmic scale; (**e**) electroluminescence (EL) spectrum under 300 mA injected current. Inset shows photo of LED under operation.

Self-assembled N-polar c-GaN NWs with core-shell InGaN/GaN multiple quantum wells (MQWs) were grown catalyst-free by MOCVD on nitridated c-sapphire substrates. NWs were grown with a low V/III ratio (~50), a high flux of silane (~200 nmol/min) under high temperature (1050 °C) and high pressure (800 mbar) [41,42]. The vertical growth of the n-doped GaN segment (~10 μm length) was promoted by the high flux of silane thanks to the formation of an ultrathin passivation layer of SiN_x_ around the stem of the GaN core [43,44]. This long, heavily n-doped segment was elaborated to avoid short-circuiting the device with the bottom contact. The silane addition was then stopped, and axial growth continued to form the unintentionally doped GaN (u-GaN) wire (~15 μm) with a rather strong residual doping of about 10^18^ cm^−3^ [45]. This step was followed by the radial growth at 400 mbar of seven InGaN multiquantum wells at 780 °C (5 nm thick within content of 12 ± 2%) separated by GaN barriers (30 nm thick) grown at 885 °C around the GaN wires. The active heterostructure was only formed around the upper part of the wire, while the heavily doped bottom part remained free of any deposition thanks to the passivating SiN_x_ layer on its surface [42,44]. Lastly, a 70 nm thick p-doped GaN shell was grown at 920 °C, followed by dopant activation annealing at 700 °C for 20 min. Figure 1a shows the SEM images of a typical as-grown sample. Average wire density was estimated to be around 10^6^ per cm^2^, while the diameter and the length of the wires were in the ranges of 0.65–1.2 μm and 25–30 μm, respectively.

### 2.2. Flexible InGaN/GaN Nanowire LED Fabrication 

The fabrication procedure of flexible NW LEDs is schematically illustrated in Figure 1c. First, a thin layer of Ni/Au (4/4 nm) was deposited on the p-GaN shell, while the NW n-doped base part was protected with a photoresist layer. Then, the sample was annealed in an oxygen atmosphere at 450 °C to improve the ohmicity of the contact to the p-GaN shells. Next, NWs were embedded into PDMS by spin-coating at 5000 rotations per minute for 180 s, which yielded a thickness of around 25 µm. (PDMS was prepared with a liquid-PDMS-to-curing-agent weight ratio of 10:1) The PDMS was reticulated on a hot plate at 80 °C for 1 h. Lastly, the PDMS membrane with the NWs embedded inside was mechanically peeled off with the help of a microscalpel, and bottom-up-flipped from the sapphire substrate on an arbitrary substrate for n-contact deposition [18,19]. Ti/Al/Ti/Au (10/20/10/200 nm) was deposited as the bottom contact to the n-doped NW base parts. Then, the PDMS membrane was flipped again and mounted from the back contact side on a dedicated flexible carrier for mechanical support. The PDMS excess on the front side was etched by reactive-ion etching using CF_4_ and O_2_ gases. Then, Ag NWs dispersed in isopropanol solvent were spin-coated to form the flexible and transparent top contact. Thermal annealing at 200 °C for 20 min was applied to increase the optical transparency and the electrical conductivity of the Ag NW mesh. After annealing, this type of Ag NW mesh exhibited high transmittance of above 80% and low resistance of only 18 Ω/sq [18]. A mixture of Ag NWs of different dimensions were used to improve contact performance: short and thin Ag NWs wrapping the shells of NW LEDs provided good contact between Ag NWs and individual NW LEDs, while long and thick Ag NWs facilitated long-range current spreading. Figure 1b shows the SEM image of the annealed Ag NW network spin-coat on PDMS/nitride NW LEDs to form the top contact to Ni/Au-coated p-GaN NW shells. To improve the current spreading, a silver paint frame was added at the LED edges. The flexible LED fabricated for this study had a square surface of 1 × 1 cm^2^. This large area was chosen to respond to the needs of interior-lighting design, exploring solutions for extended curvilinear light sources.

## 3. Results and Discussion

### 3.1. Flexible InGaN/GaN Nanowire LED Properties

First, the current–voltage (I–V) curve of the flexible LED was measured as shown in Figure 1d. It presented diodelike behavior, but significant leakage current was observed, as can be seen in inset displaying the I–V curve in logarithmic scale. This leakage can be attributed to the presence of irregular parasitic crystals formed during NW growth, which were highly defective and had diameters of up to several tens of microns. We note that leakage could be reduced for LEDs with dimensions of several hundreds of microns since parasitic defects could be avoided, however, in large-area devices, leakage is systematically observed. This problem can be solved by using a selective area-growth approach with dedicated substrate prepatterning, which eliminates these growth defects and allows for low leakage currents [46]. The I–V curve also showed a reduction in the forward resistance of the LED, which was related to the nonohmicity of the contact between the Ag nanowire network and the p-GaN shell of the NWs. 

The electroluminescence (EL) signal appeared around 3.5 V (corresponding to 100 mA injected current). A room-temperature EL spectrum collected under 300 mA injection current is shown in Figure 1e with a photo of the LED under operation. The EL was dominated by a peak at 405 nm attributed to the emission of core/shell MQWs. 

A second weak contribution to the EL was visible at 480 nm, probably arising from the c-plane QWs on the NW tops, which contained more In and were subject to the internal electric field present on the polar facets [18,47,48,49]. The main EL peak showed an important spectral broadening of about 150 meV related to intrawire and wire-to-wire In fluctuations. More details about the origin of the EL contributions can be found in [47]. EL was not homogeneous over the LED surface, but presented some intensity fluctuations associated with inhomogeneities of the NW properties, which led to current hot spots. Reduction in intensity was observed in the central region of the LED, which could be attributed to the inplane resistivity of the Ag NW top contact favoring higher injection close to the conductive frame.

### 3.2. Thermal Imaging of Flexible InGaN/GaN Nanowire LED under High Current

**Figure 2:**
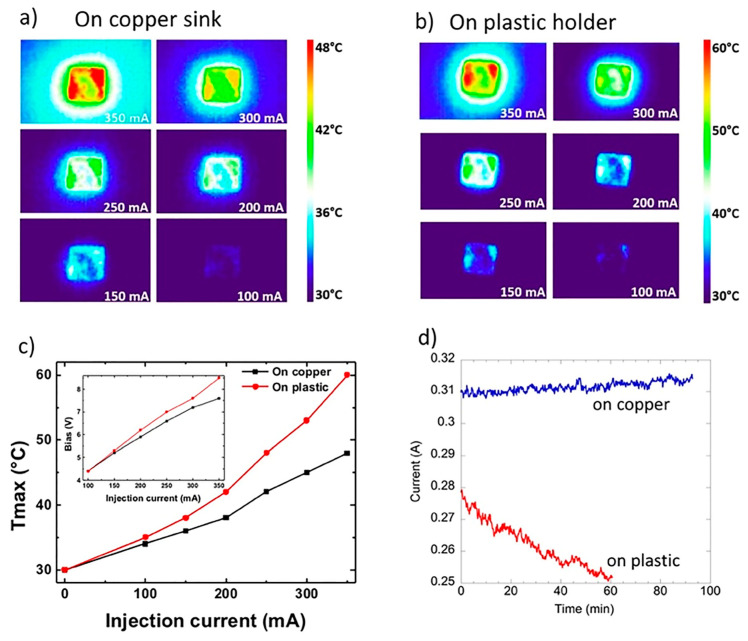
IR camera images of NW LED operating under different injection currents mounted on (**a**) copper heat sink and (**b**) plastic holder; (**c**) evolution of maximal temperature with injected current on two holders (inset shows bias for same injected current depending on holder type); (**d**) variation of current with time when forward LED bias was fixed at 7 V for the two holders.

For thermal studies, the flexible LED was mounted flatly on two different holders: a 4 mm thick copper plate and a 3 mm thick plastic holder. The first situation was considered providing a good heat sink via the copper plate, which was, in turn, mounted onto a massive metal holder. The second was considered a bad heat-dissipating medium since plastic thermally isolated the LED from the holder. Since the EL broadening prevented us from using the emission wavelength as a probe of the junction temperature, infrared imaging was used to monitor the LED heating. We measured the temperature distribution of the device surface by using an infrared camera as described in [28] for injected current increased by steps of 50 mA from 100 to 350 mA. Thermal maps of the LED and the surrounding background had spatial resolution of ~150 µm per pixel. IR images were taken after 3 min of operation at a given current. Measurements were first performed on a copper holder and then were repeated when the LED was attached to a plastic holder. Figure 2a,b show thermal maps of the flexible NW LED under increasing injection current on the copper sink and the plastic holder, both measured by the IR camera; the maximal temperature for both situations is reported in Figure 2c. With the injected current increased from 100 to 350 mA, the maximal temperature of the flexible NW LED increased from 33 to 48 °C on the copper sink, and from 35 to 60 °C on the plastic holder. This means that, for the maximal current, temperature on plastic was 25% higher than that on copper. The thermal maps showed nonuniform temperature distribution with a higher temperature close to the LED edges, which reproduced the inhomogeneity observed in the EL map discussed above (Figure 1e). The central region, which was less injected, had lower temperature due to lower Joule heating. Surface temperature was less homogeneous on plastic than it was on copper.

Figure 2c shows the maximal temperature and biases as a function of the injected current of the flexible LED on the two different holders. At the same injected current, the plastic holder bias was higher than the copper sink bias, and this difference increased with the injected current. This additional energy cost on the plastic holder corresponds to the bias difference at the same injected current, which correlated well with the temperature increase on the plastic. 

After taking thermal images, the time evolution of the electrical behavior of the LEDs under high injection was followed as presented in Figure 2d. The LED was operated under 7 V forward bias, first for 90 min on a copper holder and then 60 min on a plastic holder. The current remained almost constant over 90 min of operation on a copper heat sink. However, when the LED was mounted onto plastic, progressive current reduction was observed with a 13% decrease over 1 h. The operation was then stopped to avoid irreversible damage to the device.

All these experiments indicate that, despite the poor thermal conductivity of the PDMS matrix, a heat sink on the back side is efficient to cool down the flexible NW LED. This is not a straightforward result. Indeed, if we take the volume fraction of nitride NWs, which is below 5%, the PDMS thermal conductivity of ~1.5 W m^−1^ K^−1^, and the thermal conductivity of GaN of ~200 W m^−1^ K^−1^ [22], the average weighted thermal conductivity of the composite membrane is rather low (~11 W m^−1^ K^−1^), and backside cooling is not expected to be efficient. However, due to the strong anisotropy of the medium, cooling becomes possible, as confirmed by finite-element simulations discussed in the next section.

### 3.3. Modeling of Heat Dissipation in Flexible NW LEDs

To better understand how heat is dissipated from the flexible LED to the heat sink, the temperature distributions inside the device were simulated using 3D finite-element analysis with the Comsol Multiphysics software heat-transfer module [50]. Steady-state temperature field distribution was calculated, and material parameters were taken from the Comsol Multiphysics standard material library. In particular, the parameters for bulk GaN were used to simulate the NWs. It should be noted that modifications of thermal properties were theoretically predicted [51] and experimentally observed [52] in GaN NWs compared to bulk. However, these modifications took place for NW diameters smaller than the phonon mean free pass, which is around 100 nm in GaN at room temperature [53]. Since the diameter of the wires considered in our work is ~1 µm, bulk parameters are applicable. 

**Figure 3:**
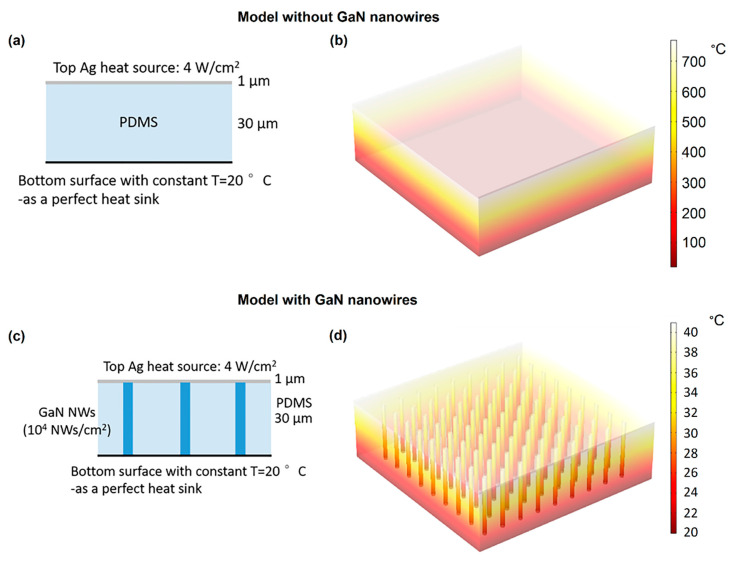
(**a**) Schematic of reference system without nitride NWs; (**b**) calculated temperature distribution in reference system; (**c**) schematic of flexible NW LED; (**d**) calculated temperature distribution in flexible NW LED.

The complex real system where heat was generated in a distributed way was replaced by a simplified model illustrated in Figure 3. The device was mounted on a perfect heat sink. The top contact was represented with a thin Ag layer and the nitride NWs were modeled as 1 µm thick GaN cylinders evenly distributed in a square lattice, with their density varying. For simplicity, we neglected injection inhomogeneities that manifested themselves as hot spots in the experimental EL distribution. Although the current hot spots caused by device imperfections may be important for device failure, their consideration is beyond the scope of the present analysis. Temperature of the bottom surface was fixed at 20 °C and kept constant.

We also made a simplifying approximation assuming that heat was generated only at the top surface of the device and not inside the NWs. This assumption may be justified by the fact that the n-doped NW core part is highly conductive, so that the heat is mainly produced close to the NW top in the active core/shell region and at the contact. In particular, contact resistance between Ag NWs and the p-GaN shell of the NWs was significant, resulting in important heat generation at this position. In the model, the top surface Ag layer was considered to be a heat source of 4 W/cm^2^ (this value corresponds to the experimentally measured power density at an injection current of 350 mA). 

First, a reference system was simulated that consisted of a 30 µm thick PDMS layer without any nitride NWs (Figure 3a). As shown in Figure 3b, which presents the calculated temperature distribution, the maximal temperature at the surface in this case was very high, reaching above 700 °C. This is a direct consequence of poor thermal conductivity of PDMS, which could not dissipate the generated heat to the bottom heat sink.

Then an array of GaN NWs connecting the top Ag heat source and the bottom heat sink was embedded in the PDMS membrane with a density of 10^4^ cm^−2^, as illustrated in Figure 3c. From the temperature distribution shown in Figure 3d, it was found that the maximal temperature at the top surface in this case was significantly lower than that without the NWs (it was equal to about 41 °C at the surface). Due to the strong simplifying assumption, this basic model is not expected to yield a quantitative explanation of the experiment results, but it does demonstrate the feasibility of cooling the flexible NW LED by using a heat sink on the back side. Indeed, nitride NWs were efficient to release the heat from the top contact of the flexible LED to the bottom heat sink, which cannot be done directly through a thermally resistive PDMS membrane.

## 4. Conclusions

The self-heating behavior of flexible InGaN/GaN NW LEDs was analyzed by IR imaging. We showed that, despite the poor thermal conductivity of the polymer matrix, active nitride NWs were efficient in dissipating heat to the substrate. Therefore, they could be cooled with a dedicated flexible heat sink integrated on the bottom side. When operated on a copper holder, the flexible LED showed stable behavior for 90 min operation under high injection, while current decrease was observed for an uncooled LED. Modelling of the temperature distribution in a NW/polymer composite membrane demonstrated that the nitride NWs were very efficient in dissipating heat from the LED.
